# On closed-loop brain stimulation systems for improving the quality of life of patients with neurological disorders

**DOI:** 10.3389/fnhum.2023.1085173

**Published:** 2023-03-23

**Authors:** Abdelkader Nasreddine Belkacem, Nuraini Jamil, Sumayya Khalid, Fady Alnajjar

**Affiliations:** ^1^Department of Computer and Network Engineering, College of Information Technology, UAE University, Al-Ain, United Arab Emirates; ^2^Department of Computer Science and Software Engineering, College of Information Technology, UAE University, Al-Ain, United Arab Emirates; ^3^Center for Brain Science, RIKEN, Saitama, Japan

**Keywords:** brain stimulation, closed-loop BCI, neurodegenerative diseases, psychiatric diseases, brain computer interface (BCI)

## Abstract

Emerging brain technologies have significantly transformed human life in recent decades. For instance, the closed-loop brain-computer interface (BCI) is an advanced software-hardware system that interprets electrical signals from neurons, allowing communication with and control of the environment. The system then transmits these signals as controlled commands and provides feedback to the brain to execute specific tasks. This paper analyzes and presents the latest research on closed-loop BCI that utilizes electric/magnetic stimulation, optogenetic, and sonogenetic techniques. These techniques have demonstrated great potential in improving the quality of life for patients suffering from neurodegenerative or psychiatric diseases. We provide a comprehensive and systematic review of research on the modalities of closed-loop BCI in recent decades. To achieve this, the authors used a set of defined criteria to shortlist studies from well-known research databases into categories of brain stimulation techniques. These categories include deep brain stimulation, transcranial magnetic stimulation, transcranial direct-current stimulation, transcranial alternating-current stimulation, and optogenetics. These techniques have been useful in treating a wide range of disorders, such as Alzheimer's and Parkinson's disease, dementia, and depression. In total, 76 studies were shortlisted and analyzed to illustrate how closed-loop BCI can considerably improve, enhance, and restore specific brain functions. The analysis revealed that literature in the area has not adequately covered closed-loop BCI in the context of cognitive neural prosthetics and implanted neural devices. However, the authors demonstrate that the applications of closed-loop BCI are highly beneficial, and the technology is continually evolving to improve the lives of individuals with various ailments, including those with sensory-motor issues or cognitive deficiencies. By utilizing emerging techniques of stimulation, closed-loop BCI can safely improve patients' cognitive and affective skills, resulting in better healthcare outcomes.

## 1. Introduction

Until recently, controlling one's environment through mental activity or by sending information to the human brain was only an artifact of science fiction. However, recent advances in brain computer interface (BCI) technology have turned this into reality. BCI allows humans to exchange information with their environment by using the electrical signals of brain activity to control any external device. It is a rapidly emerging field for developing integrated software–hardware systems that enable users to send real-time neural commands to any external device *via* the Artificial Intelligence-based interpretation of brain activity. The BCIs system creates an information pathway between the brain and the world by interpreting the relevant patterns of neural activity during cognitive or affective brain processes. Further, it allows for bidirectional information flow by reading and sending information to and from the brain. BCI applications aim to support, enhance, and restore human cognitive abilities, such as sensorimotor functions (Krucoff et al., 2016). In layman's terms, BCI enables people to control machines with their thoughts. It can empower people who are incapable of speaking, seeing, hearing, or moving their limbs to directly communicate with computers by essentially bypassing the normal central nervous system (CNS) pathway by using only their brain activity. Thus, closed-loop BCIs are changing the concept of restoration and rehabilitation by linking neural activity with the environment, and providing the external regulation or the self-regulation of brain functions by using many types of feedback. For example, closed-loop BCI has various applications in advanced neuroprosthetics (Pan et al., 2020) and neurofeedback training/therapy (Lotte, 2012). These closed-loop BCI systems enable reading and writing from and to the CNS, and are vital for treating neurological disorders, movement disorders, epilepsy, and memory disorders as well as for stroke rehabilitation (Lee et al., 2019). They act on central and peripheral structures, such as the cranial nerves (vagus) (Uthman et al., 1993; Ben-Menachem et al., 1994; Tatum and Helmers, 2009; Ogbonnaya et al., 2013; Johnson and Wilson, 2018); and cortical (Morrell, 2011; Heck et al., 2014; Sun and Morrell, 2014; Lee et al., 2015) and subcortical structures (Salanova et al., 2015) of the brain.

Standard BCIs can be broadly classified according to electrode placement as (1) non-invasive, (2) partially invasive, and (3) invasive. Non-invasive BCIs record signals from electrodes placed on the scalp, e.g., electroencephalography (EEG) (Wolpaw et al., 2002; Wolpaw and McFarland, 2004). Partially invasive BCIs involve electrodes planted inside the skull *via* craniotomy but external to the brain [e.g., intracranial EEG (iEEG); Wang et al., 2016]. Invasive BCIs use microelectrodes directly placed into the gray matter to capture the signals from neurons [e.g., electrocorticography (ECoG); Milekovic et al., 2012; Hammer et al., 2013, 2016]. By using EEG decoding, synchronous and asynchronous control and communication are established by the BCIs. These non-invasive neural systems or EEG-based BCIs are further based on two categories of brain activity: “evoked” and “spontaneous.” In “evoked” BCIs, the brain generates an immediate automated response to an external stimulus. In “spontaneous BCIs,” the EEG records brain activity associated with mental tasks performed according to the user's volition. For example, P300 and the steady-state visually evoked potential (SSVEP) are based on the “evoked” potential (Chamola et al., 2020). By contrast, motor imagery (MI) is the process by which an individual stimulates a physical reaction *via* mental stimulation (Pfurtscheller and Neuper, 2006). Another method of classifying BCI is based on the presence or absence of opened-loop and closed-loop feedback. Open-loop adaptive systems do not involve user feedback, and use measurements of the state of BCI users as an implicit input to execute adaptation without giving them the right to correct/adjust their actions. By contrast, closed-loop BCI is an adaptive system that uses simple or complex neurofeedback to analyze brain processes and initiate neuroplasticity, or modulate and enhance brain activity by using techniques of brain stimulation. These techniques have been used for many therapeutic applications [e.g., cranial electrotherapy stimulation (CES), deep brain stimulation (DBS), transcranial direct-current stimulation (tDCS), electroconvulsive therapy (ECT), low-field magnetic stimulation (LFMS), functional electrical stimulation (FES), magnetic seizure therapy (MST), vagus nerve stimulation (VNS), deep transcranial magnetic stimulation (Deep TMS), and responsive nerve stimulation (RNS)].

Therefore, a technology known as closed-loop brain-computer interface based brain stimulation has the potential to be utilized in a wide variety of medical contexts. It has the potential to completely change how neurologists, psychiatrists, and other medical professionals diagnose, treat, and manage neurological and mental health conditions. It is essential to do research on this technology to get an awareness of the advantages and disadvantages it may present, as well as to identify the most effective applications for it. Patients suffering from neurological and mental health conditions may be able to speak with the help of BCI-based brain stimulation, which is one of the possible benefits of this type of brain stimulation. Without having to rely on verbal communication, this might make it possible for medical professionals to identify and treat the aforementioned illnesses. Patients who have impairments may be able to regain some amount of independence as a result of this treatment option.

Hence, this systematic review aims to explore the potential benefits and publication trends with close-loop BCI-based brain stimulation in neurodegenerative and psychiatric diseases. Get a comprehensive grasp of the ways in which various forms of brain stimulation influence brain function as well as behavior.

### 1.1. Closed-loop BCIs

The most advanced BCI systems use “closed-loop” strategies in which the implanted devices have in-built read–response mechanisms and embedded algorithms that automatically adjust simulation-related factors to match the patient's needs (Lee et al., 2015). These devices sense the composition of the patient and stimulate signals only when required, thereby reducing the side-effects during treatment. This can help conserve power and thus minimize battery replacement surgeries. These systems profoundly impact the clinical pathways of patients with complex nervous system diseases. This strategy has been used for mobility assistance—for example, for wheelchair control or rehabilitation purposes, such as for controlling an electrical stimulator.

**Figure 1:**
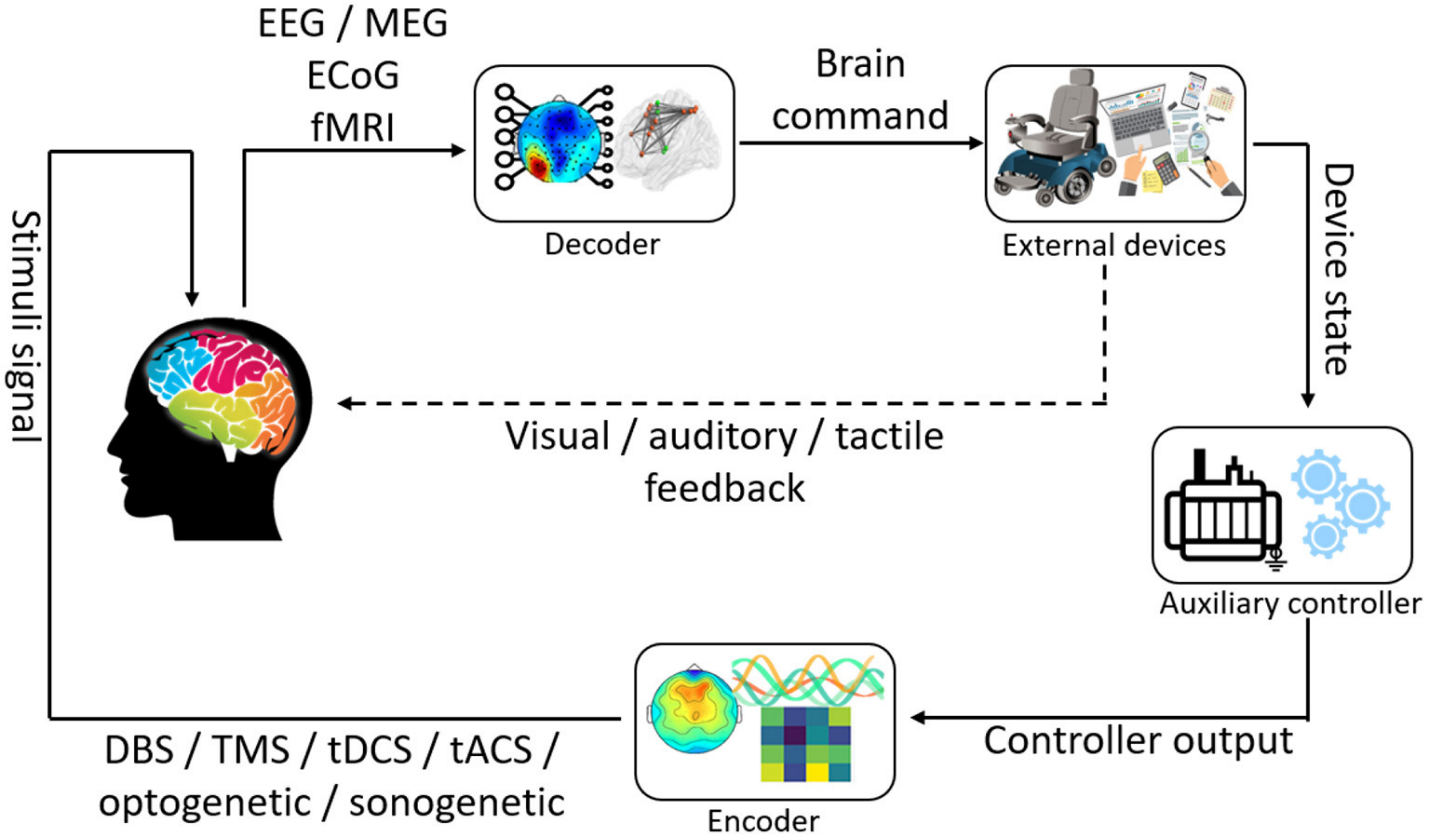
The closed-loop system based on brain stimulation with feedback.

A closed-loop BCI system usually involves a control paradigm, measurement, processing, prediction, and feedback from the application (Ahn et al., 2014) as shown in Figure 1. These functions help understand or modulate the user's mental condition or intention. The system uses these data to execute some predefined functions to interact with the environment by seeing, hearing, or sensing the action. An external device provides communication and neurofeedback, which enables the user to determine how well they can control the device. Closed-loop BCI can signal to the brain (e.g., through feedback *via* electrical/magnetic stimulation, and optogenetic and sonogenetic techniques) to correct the action or obtain additional information about it.

The control paradigm is the input provided by the BCI user to generate brain signals related to their intent. The user generates this input *via* mental tasks, including the kinematical/visual imagining of the physical movement of a body part, or by concentrating on a specific object to generate a P300 wave. Some BCI systems (e.g., affective BCI) do not require the users' intentions. Still, they function by identifying the emotional and mental statuses of the user, and can be active, reactive, or passive (Gürkök et al., 2012). In any case, these generated signals must be measured *via* invasive or noninvasive techniques. Invasive techniques, including ECoG, microelectrode arrays, and single microelectrodes, can detect signals from the brain's surface and produce high-quality signals. However, these techniques require risky implantation surgeries.

Therefore, BCI research commonly uses noninvasive methods to detect signals (e.g., magnetoencephalography and functional-magnetic resonance imaging). EEG is the most popular and preferred technique in this regard (Nicolas-Alonso and Gomez-Gil, 2012) as it is economical and portable, and can even be measured by using wireless devices. The most popular closed-loop BCI is the one based on motor imagery, where the disabled can receive a piece of additional sensory information from the BCI device. This BCI-based control system of closed-loop brain stimulation can help sense the effect of a stimulus and adjust this stimulation in response to the observed effect. The closed-loop BCI can be used to create synaptic plasticity through spike-triggered stimulation.

BCIs allow users to communicate with external or control any external devices, such as robotic arms, with their thoughts by monitoring brain activity from the motor cortex and decoding movement intentions using machine learning techniques which leads to significantly improve the quality of life of healthy and unhealthy people such as patients with spinal cord injuries or other paralysis to restore some motor functions (Gao Q. et al., 2017; Belkacem et al., 2018, 2020; Al-Nuaimi et al., 2020; Belkacem, 2020; Shao et al., 2020; Chen et al., 2021a,b; Jamil et al., 2021). Closed-loop brain stimulation can also assist motor learning and enhance the recovery of motor function in stroke patients and those with motor deficits (Xu et al., 2013).

BCIs and closed-loop brain stimulation can be used to treat a variety of neurological and psychiatric conditions, such as Parkinson's disease, epilepsy, depression, and obsessive-compulsive disorder (OCD) (Liang et al., 2010; Widge and Moritz, 2016; Arlotti et al., 2021; Sani et al., 2021). For instance, in Parkinson's disease, closed-loop brain stimulation can administer electrical stimulation to the brain in response to aberrant neural activity, alleviating symptoms such as tremors and stiffness. By detecting seizure activity and applying focused brain stimulation to avoid seizures, BCIs can also treat epilepsy.

Moreover, closed-loop BCI can improve cognitive performance in healthy persons. BCIs can increase attention and working memory, for instance, by monitoring brain activity linked with these cognitive processes and giving the user feedback. By giving tailored brain stimulation during certain cognitive activities, closed-loop brain stimulation can also be utilized to improve cognitive performance (Jamil et al., 2022).

Insomnia and sleep apnea are two examples of sleep problems that can be treated using BCIs and closed-loop brain stimulation. By administering tailored brain stimulation to encourage breathing or alertness, closed-loop brain stimulation may be used to detect and respond to sleep-related events such as apnea episodes. This type of brain stimulation can be used to detect and respond to sleep-related events. BCIs may also improve sleep quality by identifying sleep-related neural activity and giving tailored brain stimulation to enhance the sleep state. This can be accomplished by monitoring the neural activity that occurs during sleep (Choi et al., 2020).

**Figure 2:**
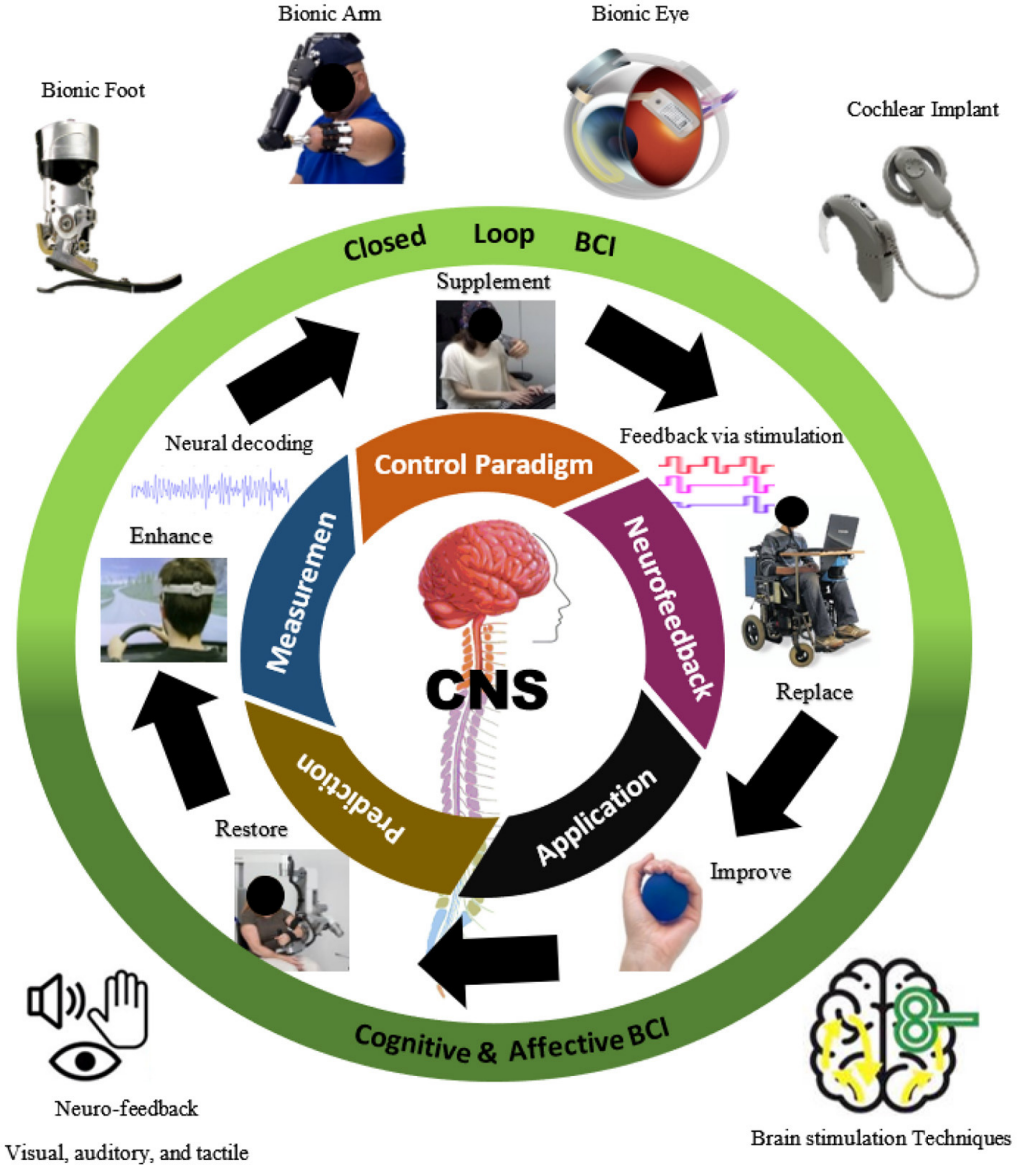
A brain-computer interface (BCI)-based control/communication of closed-loop brain feedback or stimulation: methods, applications, and effects.

The measured brain signals are then processed for the maximum signal-to-noise ratio. The selection is performed *via* certain algorithms (Bashashati et al., 2007; Roman-Gonzalez, 2012) including those for spatial and spectral filtering, to derive information from these signals, which are then used as inputs for the classification modules. The steps of prediction involve making decisions based on the user's intention or by quantifying their emotional and mental statuses. Machine learning algorithms and artificial neural networks are usually applied for prediction (Bashashati et al., 2007; Al-Ani et al., 2010). Once the user's intention has been determined, this output is used to change the environment. This change is then provided as feedback to the user. Figure 2 shows the control and communication involved in closed-loop brain feedback or stimulation: its methods, applications, and effects.

User experience and neural feedback are very important in closed-loop BCIs as they provide information regarding the success of the effort. Furthermore, neurofeedback allows users to improve BCI control through self-regulation and learning. In neurorehabilitation, this feedback is linked to neuroplastic stimulation that can be used to modify and modulate the user's neural activity. Neurofeedback training is the conscious alteration of brain signals by the user (Boyd et al., 2017). The BCI user learns to control their brain activities based on measurements of the brain activity and feedback signals. For example, in case of diseases like stroke, the rhythm of the brain slows down and there is a substantial decrease in cortical activity, resulting in motor and cognitive impairments (Boyd et al., 2017; Kim and Winstein, 2017). Neurofeedback training aims to restore these brain signals to facilitate a more standard state, possibly leading to functional recovery (Remsik et al., 2016). Neurofeedback can be visual, auditory, and tactile. It can be used for various ailments, including depression, anxiety, stress, pain, attention deficit hyperactivity disorder, cognitive impairments, insomnia, schizophrenia, and motor recovery.

### 1.2. Neurofeedback

Neurofeedback, is a form of biofeedback, a noninvasive therapeutic approach that detects a patient's brain activity and delivers real-time feedback regarding how the brain works. Neurofeedback treatment is used to train patients to control their brain processes by showing them how the brain reacts to certain stimuli. It involves analyzing brain activity and offering instant feedback, frequently *via* visual or audio signals.

Attention deficit hyperactivity disorder (ADHD), anxiety, depression, and post-traumatic stress disorder (PTSD) have all been treated with neurofeedback, a therapy method that uses real-time brain activity displays to teach people how to regulate their brain activity. Several studies have shown that neurofeedback can assist people with these diseases manage their symptoms and improve their quality of life. For instance, a meta-analysis of trials looking at neurofeedback for ADHD discovered that it significantly reduced hyperactivity/impulsivity and improved attention, which effects persisted over time (Arns et al., 2009). It is important to note, though, that not all studies have found neurofeedback to be effective, and some have found its effects to be either temporary or restricted (Sonuga-Barke et al., 2013).

Auditory feedback (AF) is any audible output that helps the user interact better with the system. It is used during speech learning in infants. AF can be used in BCI training by manipulating the audio input to achieve the target outcome. Two commonly used manipulations are delayed auditory feedback (DAF) and altered auditory feedback (AAF). In DAF, there is no change in the auditory signal, but it is sent to the listener after a short delay. In AAF, some sections of the signal, such as its pitch or structure, are manipulated and then sent back to the listener without delay. DAF has been shown to improve fluency among people who stutter (Yates, 1963; Ryan and Van Kirk, 1974). With regard to AAF, shifts in the pitch and other parameters in the direction opposite to that of vocal compensation occur automatically, with no conscious awareness, in neurologically healthy speakers (Houde and Jordan, 1998; Liu et al., 2011).

Visual and auditory feedback has been used for many BCI modalities (P300 speller, SSVEP, and motor imagery). It can easily cause brain fatigue, dizziness, nausea, and other adverse reactions. Most visual BCIs are based on flickering stimuli, and continuous flickering can cause visual fatigue and reduce the user's comfort. Auditory BCI is not widely used due to its susceptibility to environmental interference and relatively low accuracy. In addition, it cannot protect the user's privacy from surrounding systems. Tactile feedback is an alternative with many advantages over visual/auditory feedback, such as the ability to generate ideal target signals without repeated training. For instance, BCI-based MI (MI-BCI) allows users to communicate *via* the imaginary movements of their extremities by using a computer.

Although MI-BCI represents a promising strategy for control, it uses visual feedback to teach the user about the system's decisions. This makes it challenging to use with visually interactive tasks. Indeed, MI-BCIs have rarely been used outside the laboratory (Jeunet et al., 2015) dowing to their flawed classification algorithms and the difficulty of sight-based learning. These mechanisms simulate the sensation of tapping as a response to touch. The response is realized *via* vibrations. However, device operation is not interrupted by tactile feedback (Lukoyanov et al., 2018).

Several studies have explored tactile (vibration) stimulation in MI-BCI (Cincotti et al., 2007; Leeb et al., 2013; Gwak et al., 2014). The replacement of visual feedback by vibrotactile feedback does not inhibit EEG measurements in MI-BCI (Leeb et al., 2013) and thus does not negatively affect the classification accuracy (Cincotti et al., 2007; Leeb et al., 2013). However, this replacement reduces the visuomotor load during the tracking of multiple objects (Gwak et al., 2014). Thus, MI-BCI provides tactile feedback, and pays more attention to the problem and less to the feedback (Cincotti et al., 2007), to ensure a high accuracy of classification.

### 1.3. Neural prostheses (NPs)

In the event of an injury or a disease that compromises a particular area of the brain, neural prosthesis (neuroprosthetics) can be used to restore function in the affected area, whether it is motor, sensory, or cognitive. Swann et al. (2018) showed that fully implanted neural prostheses can be used to produce adaptive deep brain stimulation for patients of Parkinson's disease. The nanocognitive device called an “endomyccorhizae-like interface” (the future of neuroprosthetics) was created to improve features of the neural network in people with neurodegenerative diseases such as Alzheimer, Parkinson's, or dementia (Saniotis et al., 2018).

Cochlear implants are the most widely used among neuroprosthetics. Individuals with moderate-to-profound and severe sensorineural hearing loss (SNHL) benefit more from cochlear implants than hearing aids. Cochlear implants directly stimulate the auditory nerve, avoid injured cochlear hair cells, and offer salient coded information for better speech perception (Buchman et al., 2020). When the retina is damaged, it can cause visual problems and lead to total blindness in extreme situations. The retina is the part of the eye linked to the brain. It contains photoreceptors that generate electrical signals from light, which are then transmitted to the brain *via* the optic nerve. Many other bionic eye systems have been developed in recent years, including artificial silicon retina that uses a silicon chip containing solar-cell microphotodiodes. These photodiodes convert light energy into electrical impulses sent to the brain *via* the optic nerve (Buchman et al., 2020; Suresh, 2020).

### 1.4. Brain stimulation for neurodegenerative and psychiatric diseases

Neurostimulation is the intentional modulation of the activity of the nervous system by using invasive methods, such as microelectrode implantation, or noninvasive techniques, such as transcranial magnetic stimulation (TMS). Neurons in the brain collaborate in vast networks to regulate and coordinate activities of the body, such as seeing, listening, sensing, and feeling to perform actions, and control respiration and pulse. An electrical signal is produced by the neuron whenever it is stimulated, and can be changed through TMS by applying an electromagnetic coil to the scalp. The electromagnet provides a magnetic pulse that activates nerve cells in the mood regulation- and depression-related areas of the brain in a harmless manner. It is thought to stimulate brain areas that have diminished activity in case of depression.

Deep brain stimulation (DBS) is an invasive neurostimulation technique that requires surgery to implant a neurostimulator device that sends electrical signals to specific parts of the brain responsible for body movements. Electrodes are placed on the right and left sides of the brain, and are connected *via* long wires. They are then are placed under the skin, traveling down the neck, and are connected to a battery-powered stimulator placed under the skin near the chest (Artusi et al., 2018). The patient can use a handheld controller to control the DBS system. The stimulation settings can be adjusted per the patient's condition. DBS can be used to treat both movement-related and psychiatric disorders. Further, it has shown therapeutic success for otherwise treatment-resistant activity-related and affective disorders, such as tremors, dystonia, Parkinson's disease, chronic pain, and psychiatric disorders (such as depression, bipolar disease, obsessive–compulsive disorder, and Tourette's syndrome; Lozano et al., 2019). DBS has recently been considered to regulate action in memory circuits, where this indicates its potential for therapeutic use to treat dementia and Alzheimer's disease (Freund et al., 2009; Kuhn et al., 2015; Mirzadeh et al., 2016). Different DBS targets have been used to treat patients with Alzheimer's disease and yielded promising outcomes, including slower cognitive decline and improved functional brain connectivity (Laxton et al., 2010; Lozano and Lipsman, 2013). As the biological history of neurodegeneration cannot be reversed in humans, DBS may serve as supplementary treatment by controlling memory circuits (Lv et al., 2018). DBS transmits electrical impulses to the area of the brain responsible for movement-related symptoms caused by Parkinson's disease. Electric impulses disrupt these symptoms, resulting in abnormal activity in the brain's circuitry. In people with Parkinson's disease, three brain regions responsible for motion control are targeted by using DBS: the subthalamic nucleus, the globus pallidus internus, and the thalamic ventral intermediate nucleus. Targeting a specific brain area in this case depends on the treatable symptoms. Transcranial direct-current stimulation (tDCS) is a non-invasive technique for brain stimulation widely used in clinical trials for neurological and psychiatric disorders. It can mitigate depression by stimulating nerves of the left dorsolateral prefrontal cortex (Shiozawa et al., 2014).

Optogenetics is a new approach that combines optics and genetics to control the activity of certain neurons. The key element of optogenetics is the use of light. Many studies have implemented optogenetics in models of diseases, such as epilepsy, Alzheimer, Parkinson's, sensory system degeneration, and depression.

Epilepsy is a prevalent neurological illness marked by seizures. Tønnesen et al. (2009) demonstrated that light-induced halorhodopsin activity can suppress epileptiform activity while hyperpolarizing the primary neurons of the hippocampus. The optogenetic activation of hippocampal neurons at 10 or 20 Hz induces seizure-like after-discharges in rats given doses of ketamine and xylazine as anesthesia (Osawa et al., 2013). Furthermore, Paz et al. (2013) made closed-loop devices that can stop seizures by stimulating the brain with light in real time.

With regard to Alzheimer's disease, Wang et al. (2019)demonstrated that optogenetics can be utilized to modulate the neuronal-glial network to improve memory in mice with Alzheimer's disease. In addition, optogenetics has been used to analyze the activation of a particular neural circuit in transgenic mice with the amyloid precursor protein (APP) to determine the causal relationship between synaptic activity and β-amyloid peptide (Aβ) disease (Yamamoto et al., 2015).

Optogenetics was used to examine the graft function and graft–host connection for Parkinson's disease (Steinbeck et al., 2015). Moreover, Magno et al. (2019) revealed dopamine-depleted male mice benefit from optogenetic stimulation of the secondary (M2) motor cortex in case of Parkinson's. Fougère et al. (2021) claimed that the optogenetic stimulation of glutamatergic neurons in the cuneiform nucleus improves locomotion, regulates speed, and elicits limb motions comparable to those seen in intact animals during spontaneous locomotion.

Optogenetics, in conjunction with behavioral paradigms, has frequently been utilized in rats to understand the significance of various types of neurons and their pathways in people with depression (Biselli et al., 2021). An immediate effect can be mediated in the model of depression by laser or LED light to activate the expressed channelrhodopsin to obtain the specific target of dopamine neurons in the ventral tegmental area (VTA) in mice by applying the optogenetic technique (Chaudhury et al., 2013; Tye et al., 2013). Therefore, optogenetics can be used to regulate neurons to cure neurodegenerative and psychiatric diseases.

Recent years have witnessed a rise in interest in targeted ultrasound techniques for neurodegenerative and psychiatric illnesses. Sonogenetics, which is noninvasive and has a high spatial resolution, involves the genetic manipulation of ultrasound-sensitive neurons and their unique responses to it through the development of mechanosensitive receptors. Fan et al. (2021) demonstrated the proof of concept for the therapeutic application of sonogenetics to slow neurodegeneration in animal models of Parkinson's disease. Leinenga and Götz (2015) claimed improvements in Alzheimer's memory tests by using repeated scanning ultrasound treatments on the mouse brain to eliminate Aβ, without requiring any extra therapeutic agent.

Sonogentics technology that combines deep penetration with a regionally focused ultrasound has evolved, with major therapeutic applications to seizure, depression, and Parkinson's disease (Leinenga et al., 2016). Duque et al. (2021) experimentally stimulated neurons in a mammalian brain by using ultrasound and observed the changes in behavior. Ultrasound is one of the effective tools in physiotherapy, surgery, chemotherapy, medication administration, and sonography (Mason, 2011). Neurostimulation improves the quality of life of patients with severe paralysis, sensory loss, and chronic pain. It plays a vital role in neuroprosthetics, such artificial organs as bionic eyes and limbs, and cochlear implants. Further, it can alter disease symptoms in cases where medications cannot be used owing to their severe side-effects. It is also an option for many movement disorders, with relatively minimal side-effects.

## 2. Methodology

This review analyzes and identifies the most recent and relevant research on closed-loop BCI for neurodegenerative and psychiatric diseases based on electric/magnetic stimulations, optogenetics, and sonogenetics techniques.

### 2.1. Research questions

The research questions (RQs) considered in this review, along with the rationales for them, are shown in Table 1.

**Table 1:** SLR research questions.

**No**.	**Research question**	**Rationale**
1.	How has the frequency of studies related to closed-loop BCI for brain stimulation evolved over time?	To determine the publishing trends of closed-loop BCI for brain stimulation literature throughout time.
2.	What is the most closed-loop BCI method based on electric / magnetic stimulation, optogenetics, or sonogenetics techniques for neurodegenerative and psychiatric diseases?	To identify closed-loop BCI, and determine how it supports, enhances, or restores functions to improve patients' daily lives.

### 2.2. Search strategy

**Figure 3:**
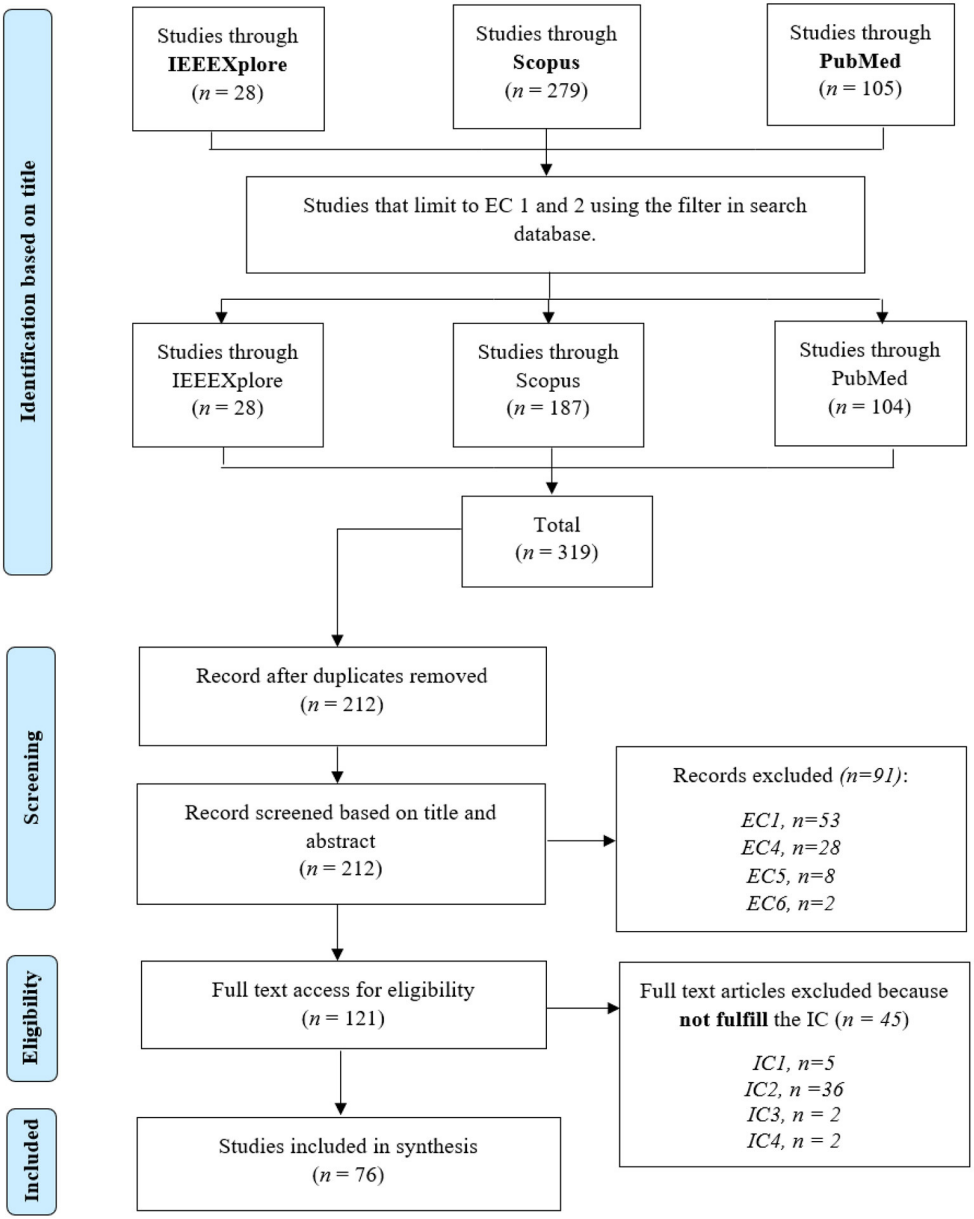
The PRISMA flowchart. EC, exclusion criteria; IC, inclusion criteria; *n*, number of publications.

This review adhered to the guideline for Preferred Reporting Items for Systematic Reviews and Meta-Analyses (Liberati et al., 2009). The following digital databases were searched: IEEEXplore, Scopus, and PubMed. The search began between early and mid-September 2022, and was constrained to the title of each paper, its abstract, and keywords to lower the number of results. The following was the primary search string used to find the relevant literature: (“closed loop BCI” OR “brain–computer interface” OR “brain–machine interface” OR BCI) AND (sonogenetic* OR optogenetic* OR “deep brain stimulation” OR “transcranial magnetic stimulation” OR “transcranial direct current stimulation” OR TMS OR DBS OR tDCS) AND (vision OR hearing OR motor OR sensory OR dementia OR Alzheimer OR Parkinson OR depression OR anxiety OR “psychiatric disease” OR neurodegenerat*). The search string was customized for each database. We eliminated duplicate publications after checking the titles of the papers. The titles and abstracts of all publications were evaluated to ensure their relevance. To establish the legitimacy of each article, we obtained and screened the complete text of all pertinent papers by using the criteria for inclusion. Figure 3 shows the process of retrieving articles for this review.

### 2.3. Selection criteria

Articles were included in this review if they met the following inclusion criteria (IC): (IC1) a primary study that used and focused on brain stimulation (TMS, DBS, tDCS, optogenetic, sonogenetic); (IC2) articles that focused on closed-loop models; (IC3) articles that concluded an improvement, enhancement, or the restoration of the brain function of the patients; and (IC4) experiments involving healthy or unhealthy participants or animals.

The exclusion criteria (EC) for this review were as follows: (EC1) articles that were non-peer-reviewed, abstracts, survey paper, and review papers; (EC2) non-English articles; (EC3) articles that focused only on EEG, MEG, ECoG, or fMRI; (EC4) articles that focused on improving or comparing machine learning or some algorithm; (EC5) articles related to ethics or organization; and (EC6) articles that could not be retrieved in full.

### 2.4. Data extraction

A full-text article was obtained for each study that satisfied the inclusion criteria with the assistance of a librarian. We retrieved the features of the articles, such as the following:

- participant, which represents the kind of participants used in the experiment;
- method of brain stimulation, which is the type of neurostimulation for the closed-loop model;
- disease, which is the kind of disease on which the researchers focused; and
- type of disease, either neurodegenerative or psychiatric disease.

## 3. Results

This review included 76 articles out of 319 potential studies. When performing a string search in the digital database, EC1 and EC2 were automatically applied. Furthermore, several of the publication titles were manually removed during the screening phase.

### 3.1. How has the frequency of studies related to closed-loop BCI for brain stimulation evolved over time?

**Figure 4:**
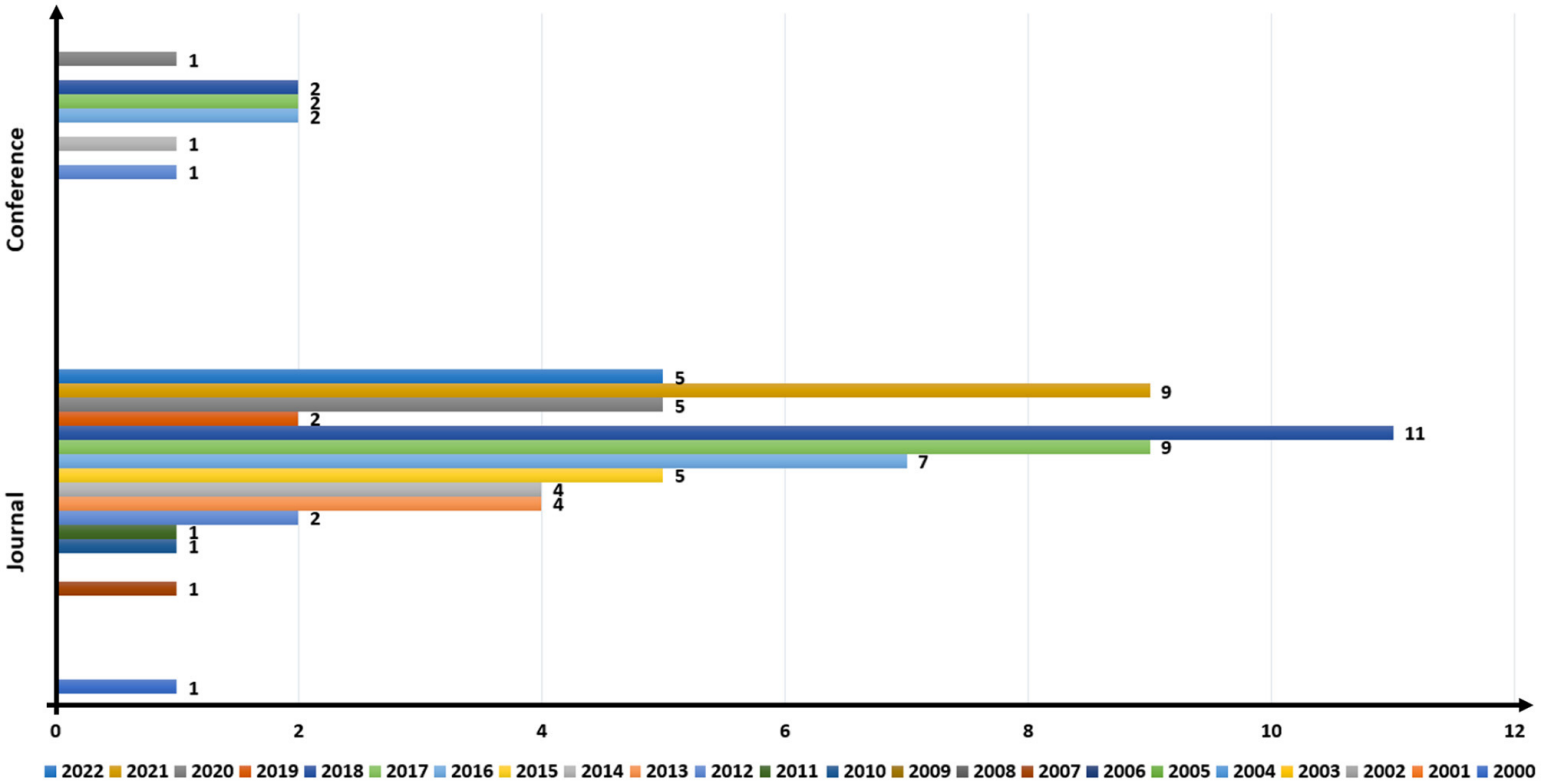
Yearly publication trend based on the research keywords.

The number of papers published annually in the area from 2000 to 2022 is shown in Figure 4. From 2000 to 2009 (excluding 2000 and 2007), there was no publication in the area. However, since 2010, the number of publications rose gradually until 2018. During 2019, the number of publications dramatically dropped to two, is likely multifactorial and complex possibilities such as changes in legislation or constraints on research may have contributed to a decline in 2019 publication totals. Regulatory bodies likely implemented tougher restrictions for closed loop brain stimulation research, resulting in a temporary decline in publications. Additionally, the advent of new study topics or a shift in research goals may have diverted resources away from closed loop brain stimulation research, resulting in a temporary decline in the number of publications. However, there was a substantial increase again starting in 2020, and the number of published papers in 2022 can increase further as the year has not ended. The largest number of published articles were from journals, with 11 papers in 2018. By contrast, the largest number of papers published in conference proceedings in any given year was two.

**Figure 5:**
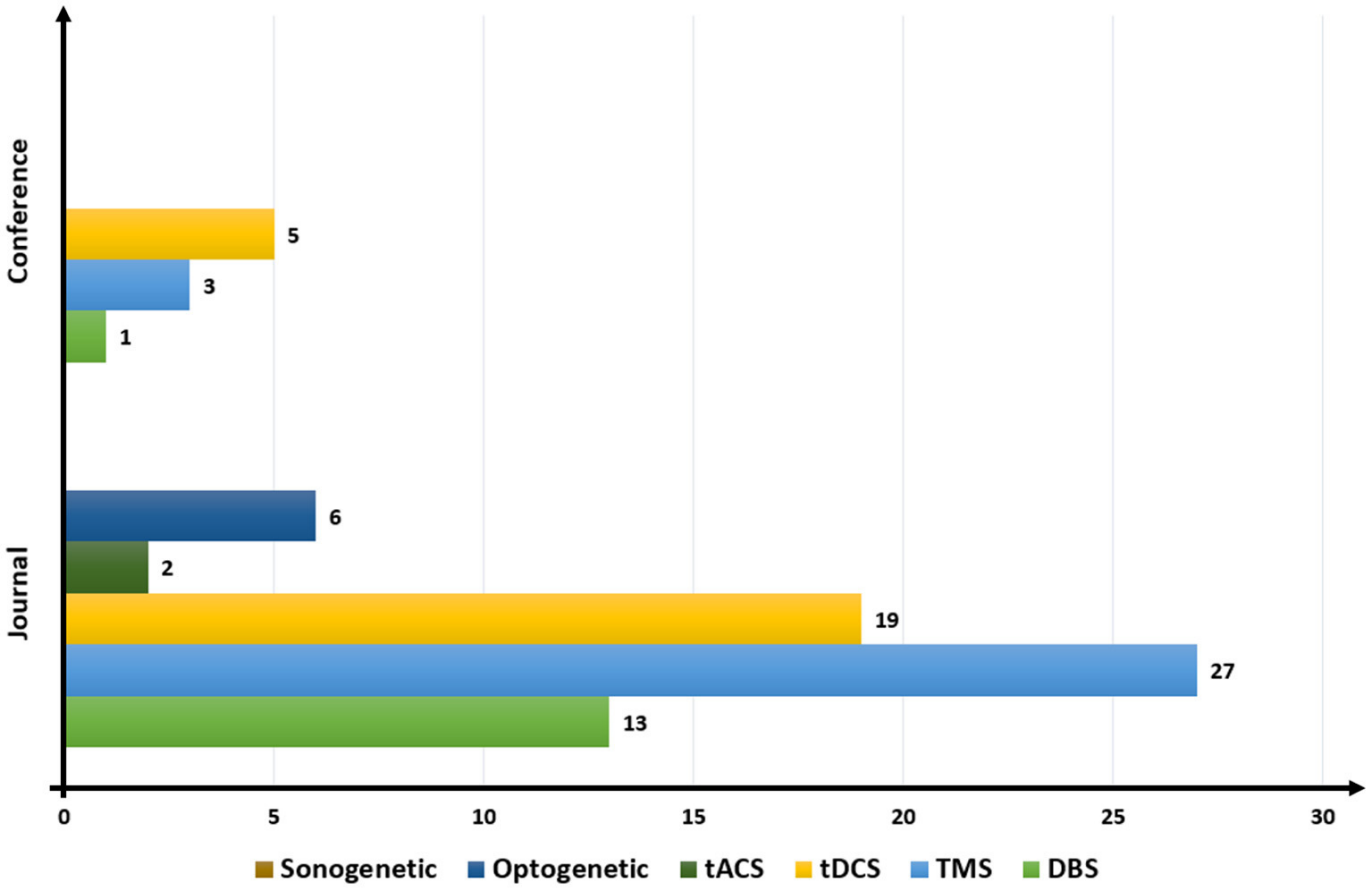
Publication trend based on brain stimulation. DBS, deep brain stimulation; TMS, transcranial magnetic stimulation; tDCS, transcranial direct current stimulation; tACS, transcranial alternating current stimulation.

Figure 5 provides information on the ratio of published papers based on the type of brain stimulation studied. The overall trend of the data shows a steep rise in the ratio of TMS in the published papers, coupled with a decline in papers on tDCS. The most highest was that 27 journals had published these articles in TMS, which is a sharp contrast with only three papers in conferences. We found no publication on sonogenetics for closed-loop BCI.

The numbers of published papers on different techniques of brain stimulation were different in conferences from those in journals. The largest number of conference papers were related to tDCS (five), followed by TMS (three). No conference paper related to tACS, optogenetics, and sonogenetics had been published. The most popular techniques of brain stimulation for closed loop BCI models from 2000 to 2022 were TMS, tDCS, and DBS.

### 3.2. What is the most closed-loop BCI method based on electric/magnetic stimulation, optogenetics, or sonogenetics techniques for neurodegenerative and psychiatric diseases?

**Figure 6:**
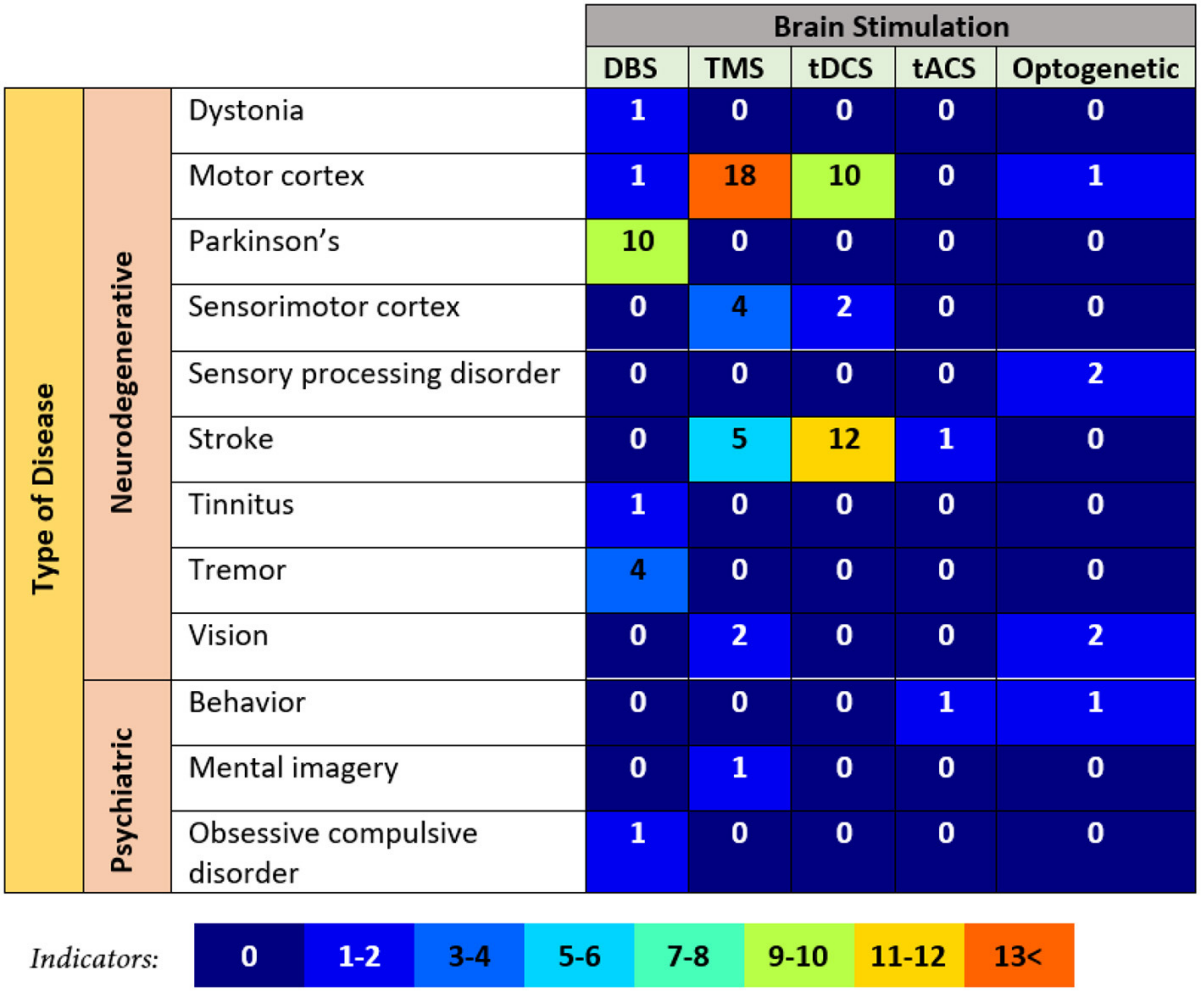
Distribution of articles selected according to the brain stimulation and type of disease. DBS, deep brain stimulation; TMS, transcranial magnetic stimulation; tDCS, transcranial direct current stimulation; tACS, transcranial alternating current stimulation.

Figure 6 depicts the distribution of articles with regard to the techniques of brain stimulation used and the types of diseases considered. They latter have been divided into the two clusters listed below:

- neurodegenerative diseases, which are related to conditions caused by the gradual destruction of cells and the nervous system synapses needed for movement, balance, muscle, sensibility, and cognition; and
- psychiatric diseases, which represent mental disorders determined by a mental health expert that significantly impair thoughts, emotions, or behavior. In general, many articles focused more on neurodegenerative diseases than psychiatric diseases, regardless of the method of brain stimulation used (Figure 6). Around 95% of all articles discussed neurodegenerative diseases and 5% discussed psychiatric diseases.

1. **Deep brain stimulation (DBS)** Among neurodegenerative diseases, Parkinson's disease was the most commonly studied by using DBS. Ten relevant articles were identified: those by Rossi et al. (2007), Little et al. (2013), Heldman et al. (2016), Swann et al. (2017), Castaño-Candamil et al. (2020), Arlotti et al. (2021), Darbin et al. (2022), Merk et al. (2022), and Neumann et al. (2021). This was followed by tremor-related disease, to which four articles were dedicated: those by Thompson et al. (2016), Herron et al. (2017), Neumann et al. (2021), and Swan et al. (2018). While Neumann et al. (2021) also discussed dystonia and tinnitus in the context of DBS. Only one article reported experiments on the motor cortex (Isaacs et al., 2000).
  Among psychiatric diseases, only obsessive–compulsive disorder (OCD) was studied by using DBS (Neumann et al., 2021) to investigate the influence of the location of implants in patients on the performance of brain-sensing devices.
2. **Transcranial magnetic stimulation (TMS)** Problems with the motor cortex have been extensively studied by using TMS, with 18 articles dedicated to the issue: those by Ros et al. (2010), Niazi et al. (2012), Sitaram et al. (2012), Mokienko et al. (2013), Takemi et al. (2013, 2018), Hänselmann et al. (2015), Kaplan et al. (2016), Royter and Gharabaghi (2016), Schildt et al. (2016), Hasegawa et al. (2017), Mashat et al. (2017), Daly et al. (2018), Jochumsen et al. (2018), Syrov et al. (2020), Ding et al. (2021), Grigorev et al. (2021), and Mihelj et al. (2021) for neugodegenerative disease. The second most commonly studied disease by using TMS was stroke, with five articles devoted to it: those by Gharabaghi et al. (2014), Syrov et al. (2019), Cantillo-Negrete et al. (2021), Hayashi et al. (2022), and Liang et al. (2020). Four articles examine the sensorimotor cortex: those by Pichiorri et al. (2011), Niazi et al. (2014), Kraus et al. (2016), and Naros et al. (2020). Vision-related diseases were investigated by two articles: those by Losey et al. (2016) and Liburkina et al. (2018).
  Meanwhile, only one article for mental imagery in the context of psychiatric disease (Vasilyev et al., 2017). It proved the correlation between psychological and neurophysiological diseases.
3. **Transcranial direct current stimulation (tDCS)** Only three neurodegenerative diseases were considered by using tDCS. Twelve articles considered stroke-related diseases: those by Ang et al. (2012, 2015), Kasashima-Shindo et al. (2015), Handiru et al. (2017), Hong et al. (2017), Hu et al. (2018, 2021), Rodríguez-Ugarte et al. (2018a), Mane et al. (2019), Chew et al. (2020), Quiles et al. (2020), and Bigoni et al. (2022). Ten articles used tDCS to study the motor cortex: those by Wei et al. (2013), Dutta et al. (2014), He et al. (2014), Soekadar et al. (2014, 2015), Takeuchi et al. (2015), Naros et al. (2016), Rodriguez-Ugarte et al. (2018), Rodríguez-Ugarte et al. (2018b), and Ortiz et al. (2020). Two articles used it to examine the sensorimotor cortex Baxter et al. (2016, 2017). By contrast, no article on psychiatric diseases used tDCS for close-loop BCI.
4. **Transcranial alternating current stimulation (tACS)** Only one article considered brain stimulation based on alternating current with a certain frequency in the context of neurodegenerative disease. It demonstrated an enhancement in self-regulation by the brain in terms of neurofeedback based on β oscillations for stroke-related disease (neurodegenerative; Naros and Gharabaghi, 2017). In the context of psychiatric disease, only one article for understanding animal behavior was used this brain stimulation (Márquez-Ruiz et al., 2016).
5. **Optogenetic** Five articles were classified into the neurodegenerative group. Two articles each were devoted to sensory processing disorders (Zhang et al., 2021; Sun et al., 2022) and vision related disease (Neely et al., 2018; Scheyltjens et al., 2018), respectively. One article considered the motor cortex, and the results showed that mice can identify neuronal activity caused by photostimuli (Abbasi et al., 2018). In the context of psychiatric disease, one article demonstrated the ability of animals to utilize an artificial cerebral channel in a behaviorally significant manner (Prsa et al., 2017).

Overall, Figure 6 shows the potential of how the closed-loop BCI based on brain stimulation systems improves the quality of life of patients. Many researchers did experiments based on the type of disease to prove that brain stimulation is one method that can restore, replace, or repair impaired brain functioning and alleviate symptoms in individuals suffering from various neurological disorders. Results have demonstrated that closed-loop DBS is superior to open-loop DBS in symptom management and in reducing adverse effects. For instance, DBS decreased the intensity of tremors in individuals who suffered from essential tremors while simultaneously decreasing the stimulation-induced adverse effects. For example, Liang et al. (2010) demonstrated that closed-loop DBS could detect and suppress epileptic seizures in real-time. Based on Figure 6 also, the selected articles show the significant clinical experiments as the practice to demonstrate the improvement in individuals with a variety of illnesses and conditions. As research into this field develops, it is anticipated that more effective and individualized treatments will become available to patients in the hope that they will have a better quality of life.

**Figure 7:**
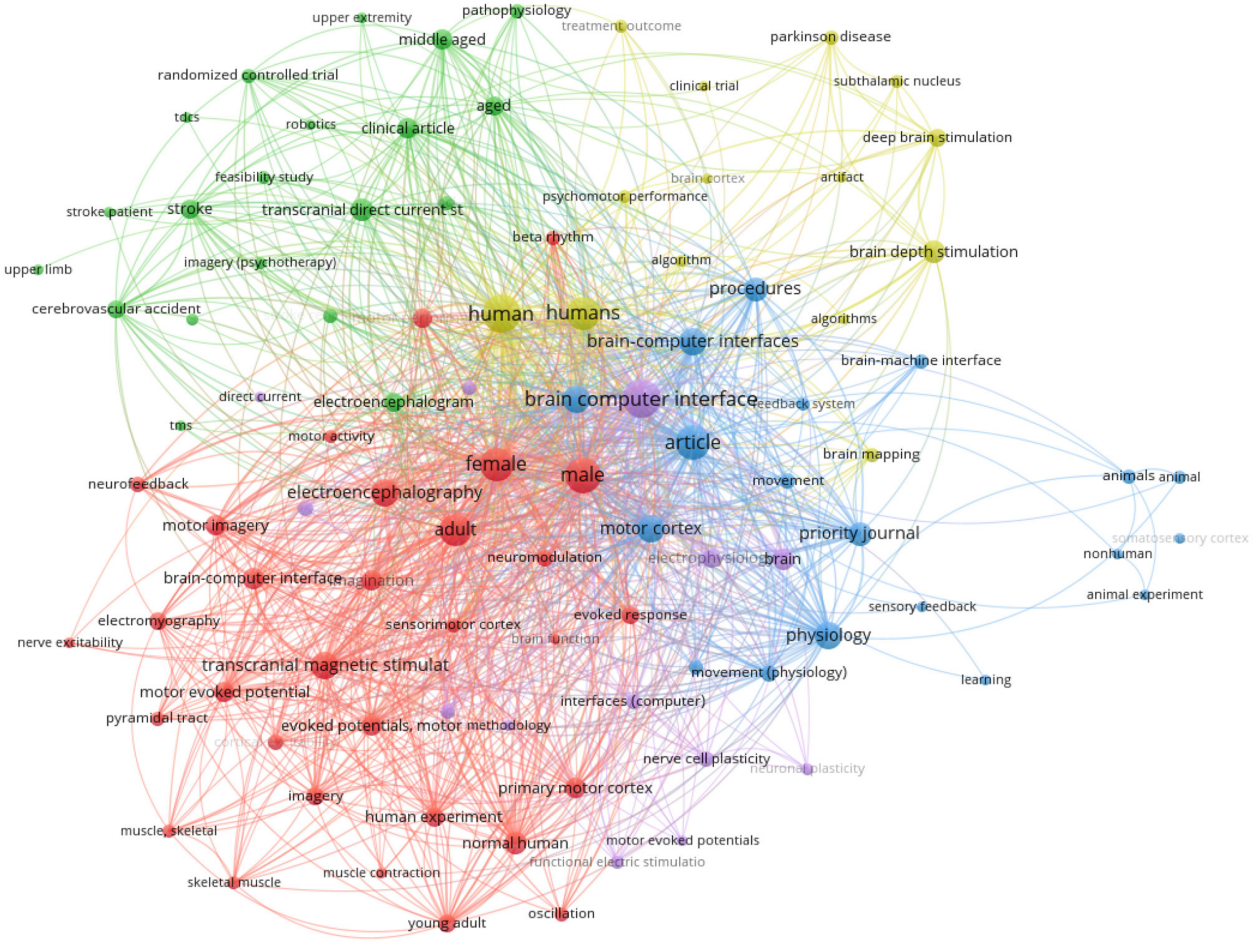
Bibliometric analysis of the appearance of keywords for closed-loop BCI based brain stimulation.

Figure 7 was constructed using the metadata from the retrieved articles. The most frequently occurring terms among the 832 keywords are human (56 times) and humans (44 times). These words must be extensively used since they constitute the foundation of the subject (patient) for experiments. This can conclude that many of researchers aims to improve the life for human who have neurodegenerative and psychiatric diseases.

Table 2 presents the mapping between the technique of stimulation used and the participants in the selected studies. Only such animals as mice or rats were used in experiments on optogenetic techniques. Healthy human participants were the most commonly considered, in 41 articles, even though they were recruited in experiments for only TMS and tDCS. By contrast, non-animal was used in TMS and tDCS brain stimulation techniques. Participants with stroke or Parkinson's diseases were the most participants in the experiments for all stimulation techniques except optogenetic.

**Table 2:** Mapping between participants and stimulation techniques identified in the selected articles.

	**DBS**	**TMS**	**tDCS**	**tACS**	**Optogenetic**	Sonogenetic*	Chemical Stimulation*
Animals	**2** Darbin et al. (2022) and Isaacs et al. (2000)	**–**	**–**	**1** Márquez-Ruiz et al. (2016)	**6** citeabbasi2018fast,zhang2021prototype,sun2022closed,prsa2017rapid,scheyltjens2018transient, and Neely et al. (2018)	**1** Zheng et al. (2020)	**2** Finlayson and Iezzi (2010) and Rountree et al. (2016)
Healthy participants	**–**	**27** Grigorev et al. (2021), Liburkina et al. (2018), Sitaram et al. (2012), Vasilyev et al. (2017), Royter and Gharabaghi (2016), Naros et al. (2020), Kraus et al. (2016), Niazi et al. (2014), and Daly et al. (2018)	**14** Baxter et al. (2017), Hong et al. (2017), Rodriguez-Ugarte et al. (2018), Soekadar et al. (2015), Dutta et al. (2014), Rodríguez-Ugarte et al. (2018a), Takeuchi et al. (2015), Soekadar et al. (2014), and He et al. (2014)	**–**	**–**	**1** Liu et al. (2020)	**–**
			Gharabaghi et al. (2014), Ros et al. (2010), Takemi et al. (2013), Mashat et al. (2017); Mokienko et al. (2013), Ding et al. (2021), Jochumsen et al. (2018), Hasegawa et al. (2017), Mihelj et al. (2021), Takemi et al. (2018), Losey et al. (2016), Niazi et al. (2012), Kaplan et al. (2016), Syrov et al. (2020), Pichiorri et al. (2011), Hayashi et al. (2022), Syrov et al. (2019), Hänselmann et al. (2015)					Rodríguez-Ugarte et al. (2018b), Wei et al. (2013), Naros et al. (2016), Baxter et al. (2016), and Ortiz et al. (2020)
Participants with disease	**12** Arlotti et al. (2021), Little et al. (2013), Rossi et al. (2007), Swann et al. (2017), Heldman et al. (2016), Herron et al. (2017); Thompson et al. (2016); Merk et al. (2022); Castaño-Candamil et al. (2020); Swan et al. (2018); Fischer et al. (2017), and Neumann et al. (2021)	**5** Sitaram et al. (2012); Cantillo-Negrete et al. (2021); Schildt et al. (2016); Gharabaghi et al. (2014), and Liang et al. (2020)	**12** Bigoni et al. (2022); Hu et al. (2021); Hong et al. (2017); Kasashima-Shindo et al. (2015); Hu et al. (2018); Handiru et al. (2017); Ang et al. (2015); Takeuchi et al. (2015); Quiles et al. (2020); Mane et al. (2019); Ang et al. (2012), and Chew et al. (2020)	**1** Naros and Gharabaghi (2017)	**–**	**1** Abbasi (2020)	**1** Pai et al. (2016)

DBS, deep brain stimulation; TMS, transcranial magnetic stimulation; tDCS, transcranial direct current stimulation; tACS, transcranial alternating current stimulation; ERD, event related desynchronization. *Denote this is not included in the selected studies but as potential brain stimulation technique identified in the most recent research without closed-loop BCI.

Table 3 shows examples of studies that focused on different techniques of brain stimulation to improve the quality of life of patients with neurodegenerative and psychiatric diseases based on close-loop BCI. We selected only one example publication for each technique of brain stimulation based on the type of publication (the top journal with the highest impact factor) and the participants involved.

**Table 3:** Potential articles sorted by brain stimulation.

**Brain stimulation**	**References**	**Participants**	**Task**	**Methods**	**Metrics**	**Result**
DBS	Herron et al. (2017)	58-year-old right-handed male with tremor	• Tasks conducted in two sets following no stimulation, open-loop stimulation, and closed-loop neural-triggered stimulation • First set of tasks was from the Fahn-Tolosa-Marin tremor assessment battery • Second set of tasks involved alternating between resting hand and bringing hand to mouth according to computer instructions.	• Electrodes were implanted on the surface of the patient's right hand motor area. • Neural signals correlated with hand movement were recorded from the cortex through these electrodes	Fahn-Tolosa-Marin (FTM) tremor assessment	Therapeutic close-loop stimulation reduced the total applied current required for movement, potentially extending the life of implanted batteries
	Darbin et al. (2022)	Two female Japanese monkeys	• A straightforward vertical hand reaching activity was employed. • The monkey was needed to approach and grasp the object with her hand within 6 s after being cued by visual and auditory cues. • The reward for completing this task successfully was a drink of water 0.1 s after touching the goal.	During the studies, surgery was undertaken to attach pipes to the skull in order to secure the head to a stereotaxic frame. • The cortical recording electrodes were placed after 10 days. • Electrophysiological mapping was used to identify the forelimb areas of primary motor cortex.	The nonparametric Kruskal-Wallis and Mann-Whitney tests were used to compare data from different situations.	Primary motor cortex γ2 adaptive DBS is a successful treatment method that requires less electrical charge supply than constant DBS to provide equivalent clinical results.
TMS	Kraus et al. (2016)	Seventeen healthy participants	• First, during motor imaging of finger extension, TMS was regulated by beta-band event related desynchronization (ERD) (16–22 Hz) and delivered inside a BCI environment. • Eleven participants serving as a control group were presented with the same quantity and pattern of stimuli when they were at rest (independent of event related desynchronization).	• During the intervention, measure electromyography (EMG) activity from the left Extensor Digitorum Communis (EDC) muscle. • Positioned two electrodes 2 cm away on the muscular belly. • Utilized a guided TMS stimulator with a biphasic current waveform linked to an eXimia Focal Bipulse Coil (5 cm mean winding diameter) to obtain MEP stimulus-response curves (SRC) prior to and during the intervention.	rmANOVA was done with Time and Intensity as “within-subject effects” and “between-subject effects”	When about 300 TMS pulses were used on the brain during beta-ERD, it caused corticospinal excitability to increase significantly and might aid in the development of novel therapeutic techniques.
	Liang et al. (2020)	Seven stroke patients	After seeing movies of wrist flexion/extension and whole-hand finger spreading, all patients were instructed to try the motions on their healthy limbs, and then envision how it would feel (kinaesthetic imagery) to use the paretic limb.	• A figure-of-eight coil coupled to a Magstim Rapid2 stimulator was used and was positioned on the healthy hemisphere above the target muscle's “hotspot” • On the stroke-affected hemisphere, the identical technique was done using the mirrored position of the healthy hotspot.	Motor evoked potential amplitudes for the first dorsal interosseous (FDI) and abductor digiti minimi (ADM) muscles above resting baseline values.	Patients with severe upper limb paralysis may use neurofeedback to increase the corticospinal excitability of the afflicted muscles.
tDCS	Baxter et al. (2016)	Twenty-nine healthy participants	• Participants were encouraged to visualize kinesthetically opening and closing their respective hand, or performing a comparable action such as squeezing a ball, independently on the target location. • Trials were terminated if the participant failed not acquire the target within 6 s.	• Installation of HD-tDCS electrodes into a 64-channel EEG cap • Calculated control signal based on a linear classifier	• Percent valid accurate (PVC) is a performance accuracy metric. • Kruskal-Wallis tests, with Wilcoxon rank-sum tests were used for *post-hoc* analysis	Electrophysiological changes in the activated sensorimotor cortex vary between left and right trials during online BCI task execution.
	Hong et al. (2017)	Nineteen stroke patients 11 healthy participants	• Each pf stroke patients participated in ten 40-min MI-BCI training sessions over a 2-week period. • Eleven healthy controls were subjected to two MRI sessions for a repeatability experiment.	The anode was put on the ipsilesional primary motor cortex, and the cathode was put on the contralesional primary motor cortex to identify the muscle of the hand was most likely to be activated.	• Fugl-Meyer assessment • Magnetic resonance imaging processing using FSL library • Voxel-wise tract-based spatial statistics	• Similar improvements were seen in motor performance, but only in the tDCS group did neuroplasticity last for a long time. • White matter integrity was improved in the ipsilesional corticospinal tract and bilateral corpus callosum.
tACS	Naros and Gharabaghi (2017)	Twenty stroke patients	All patients conducted kinesthetic motor imaging while a brain-robot interface converted β-ERD of the ipsilesional sensorimotor cortex into a robotic orthosis opening of the paralyzed hand	• Patients' paralyzed hands are linked to an electromechanical hand robot. • An autoregressive model was used to estimate the frequency power of each EEG channel. • The command signal for the brain robot interface was computed using a linear classifier based on nine characteristics. • Using Burg Algorithm	• Chi-square test • Student's *t*-test tests • ANOVA and MANOVA • An analysis of variance was used to statistically analyze behavioral and physiological data.	In compared to the baseline, intermittently-tACS enhanced the categorization accuracy of the neurofeedback intervention.
	Márquez-Ruiz et al. (2016)	Five rabbits	Classical eyeblink from the rabbit	• tACS was used to connect four silver electrodes placed over the primary somatosensory cortex. • Air-puff stimulation induces local field potentials in the vibrissa primary somatosensory cortex.	• ANOVA • Mann-Whitney test	In the associative learning paradigm, tACS of the primary somatosensory cortex vibrissa region may actually replace natural inputs during training.
Optogenetic	Zhang et al. (2021)	Twenty-four rats	The Hargreaves pain assessment toolkit's calibrated infrared (IR) generator was utilized to provide a noxious stimulus at high IR intensity and a non-noxious stimulus at low IR intensity to the hind paws of rats.	Optic fiber placement in the prelimbic prefrontal cortex and recording electrode placement in the anterior cingulate cortex.	• Student's *t*-test • Wilcoxon signed-rank test	Demonstrate the viability of using brain machine interface technology to target sensory and emotional processes linked with neuropsychiatric illnesses, both as a system for mechanistic investigation and as a therapeutic plan.

DBS, deep brain stimulation; TMS, transcranial magnetic stimulation; tDCS, transcranial direct current stimulation; tACS, transcranial alternating current stimulation; ERD, event related desynchronization.

## 4. Discussion

The results of this review show the therapeutic potential of closed-loop BCI systems for improving the quality of life of patients with neurological disorders. During our analysis of closed-loop BCI, we identified the trend of publications on closed-loop BCI within the last decade, along with the use of brain stimulation technology to enhance and improve the life of people with neurodegenerative or psychiatric diseases. The rapid growth of academic research implies that the extent and branches of techniques of brain stimulation are expanding. Closed-loop brain stimulation for neurodegenerative and psychiatric diseases has shown excellent results in clinical tests. It has the potential to enable better management of the symptoms of patients and adverse effects while using less power than is needed for standard open-loop brain stimulation (Fleming et al., 2020). Furthermore, the number of publications as well as recently discussed topics in the context of brain stimulation and closed-loop BCI show that market acceptability and empirical work in the area are rapidly increasing. Based on these findings, we may claim that the increase in scholarly work on closed-loop BCI based on brain stimulation since 2013 reflects the need for effective and safe medical treatment that can automatically modify the settings of the stimulation based on brain activity.

The results in Figure 5 indicate that the researchers' primary objective in this context is to develop novel methodologies or expand current techniques to treat neurodegenerative and mental disorders. Each technique of brain stimulation provides a different way to stimulate nerve cells of the brain. Only DBS and TMS have been approved by the US Food and Drug Administration (FDA). Therefore, many researchers have focused on them in experiments. However, DBS is a minimally invasive surgical treatment that nonetheless entails considerable risk. The insertion of the stimulator is unlikely to cause bleeding or infection in the brain (Larson, 2014). Hence, many studies have focused on noninvasive methods of brain stimulation. Even though tDSC has not yet been approved by the FDA, it is a convenient and portable method of brain stimulation that involves applying a modest amount of electric current to the scalp. Consequently, tDSC simulations accounted for the second most commonly used technique in the articles considered.

Nevertheless, the use of optogenetics and sonogenetics remains rare in closed-loop BCI. Sonogenetic stimulation is the noninvasive manipulation of neurons and other cells carrying exogenous protein channels by using ultrasound technology. However, sonogenetic stimulation is still in doubt due this technique has proved the difficulty to target the certain cells (Sato et al., 2018). Therefore, none of the studies considered here had used it for the closed-loop BCI. Few researchers have studied the viability of an auditory BCI that uses diverse EEG input signals and auditory feedback (Sellers and Donchin, 2006; Kaongoen and Jo, 2017). Transcranial ultrasonic stimulation in humans is linked with an audio distortion that can be effectively concealed (Park et al., 2021). Future directions for optogenetic and sonogenetic approaches in humans are anticipated to entail the continued development of these techniques for safe and effective therapeutic application, as well as the expansion of the variety of illnesses that can be treated with these techniques.

Optogenetics requires additional development to enhance the transport and expression of opsins in human neurons and optimize the stimulation of light sources. In addition, new opsins with enhanced characteristics and targeting capabilities must be created to allow for more precise regulation of neuronal activity. Optogenetics might be utilized to treat a wide range of neurological and psychiatric illnesses, including Parkinson's disease, epilepsy, and depression, once these obstacles are addressed. Sonogenetics requires additional development to optimize the nanoparticles used for stimulation and modify the ultrasound delivery mechanisms to ensure safe and efficient targeting of specific brain areas. Also, additional study is required to demonstrate the long-term safety and effectiveness of sonogenetics in people. Sonogenetics might be utilized to treat illnesses such as chronic pain, epilepsy, and Parkinson's disease once these obstacles are overcome. More study is required to find the optimal therapeutic uses for optogenetics and sonogenetics. This involves researching the appropriate stimulation settings, therapy duration, and patient selection criteria for these procedures.

The frequency of papers according to types of disease is shown in Figure 6. The selected articles focused on neurodegenerative diseases. Further research is needed in this context of psychiatric disease as worldwide psychological illness had risen to 13% by 2017 according to the World Health Organization (https://www.who.int/health-topics/mental-health#tab=tab_2). The results indicate that although brain stimulation supports treatment for many diseases, the main domains of concern are the motor cortex, Parkinson's disease, and stroke. Alzheimer's disease is a potential area to further explore the use of closed-loop BCI-based brain stimulation. Around 5.8 million Americans suffered from Alzheimer's in 2020. As an alternative therapy, repetitive TMS (rMTS) without closed-loop BCI has been used to treat patients with Alzheimer's disease as well (Chou et al., 2020). The clinical impacts of rTMS have been determined by using various factors to stimulate and match different cortical areas, primarily the dorsolateral prefrontal cortex. An array of advantages in cognition have been highlighted, including with language and episodic memory, behavior, and functionality, in everyday activities for patients with Alzheimer's.

Further applications and devices can be created by using closed-loop BCI based on brain stimulation. It is an integrated software–hardware system that allows the user to control an external device by using brain signals by developing information pathways to and from the brain, and responding according to the output of a given signal or stimulation. Neuroprosthetics is a rapidly emerging field that aims to develop assistive devices to fully or partially restore lost functionality owing to neuronal damage, where these devices can be external or implanted. Implanted devices generally help restore limb movement *via* electrodes placed under the skin or muscles for stimulation. Visual prosthesis, popularly known as the bionic eye, is a device intended to restore functional vision in individuals who have completely or partially lost sight. Several techniques have been proposed for stimulating the retina, including electrical stimulation (Fujikado et al., 2011), neurotransmitter stimulation (Finlayson and Iezzi, 2010), ultrasound stimulation (Jiang et al., 2018), photodiode stimulation (Lowery et al., 2017), and cortical stimulation (Tochitsky et al., 2017).

Cochlear implants are small devices that electrically stimulate the cochlear nerve to enable hearing. The external part of the device is placed outside the ear, and has a microphone that detects sounds, and then processes and transfers them to the internal part of the implant. It is surgically implanted, and provides patients with moderate-to-severe sensorineural hearing loss and a modified sense of sound. The electrical signals promptly stimulate the auditory nerve. Cochlear implants are currently the world's most successful medical prostheses, with a rejection rate lower than 0.2% and a failure rate of 0.5% (Lowery et al., 2017). In addition to neurodegenerative disease, depression can be treated by supplying repetitive magnetic pulses. During an rTMS session, an electromagnetic coil is positioned along the scalp near the forehead. A magnetic pulse is then painlessly applied to stimulate the nerve cells in the brain region responsible for mood control and depression. This treatment can help psychologists regulate the patients' behavior and mood. Table 4 shows examples of studies on brain stimulation that can be extended by using closed-loop BCI.

**Table 4:** Selected of the potential studies and their technique for bionic eye and cochlear implants.

	**References**	**Modality and method**
Bionic eye	Fujikado et al. (2016), Fujikado (2017)	- Electrical stimulation of retina - 49 channel electrodes for suprachoroidal-transretinal stimulation was implanted in the scleral pocket. - Functioning of the prosthesis was verified by behavioral tasks.
	Gao M. et al. (2017)	- Ultrasound stimulation prosthesis - A new contact-lens array transducer was proposed for use in an ultrasound retinal prosthesis - Multi-point stimulation of retina by transmitting beam- former technology to generate diverse excitation patterns.
	Finlayson and Iezzi (2010)	- Neurotransmitter stimulation of retina - Glutamate was applied locally through glass micropipettes with tip openings between 1 and 2 μm and filled with 400 μM to 10 mM glutamate dissolved in Ames medium - Two robotic micropositioners were used for recording and glutamate delivery.
	Rountree et al. (2016)	-Neurotransmitter stimulation of retinal ganglion cells (RGCs) - Glutamate stimulation using glass micropipettes - Tip were positioned near target RGCs using a micromanipulator - Once positioned at the target location, glutamate was injected using 0.69 kPs pulses from a pressure injector system.
	Lowery et al. (2017)	- Cortical implants - Tile was powered by a wireless transmitter held at the back of the head by a glass frame. - Commands were decoded from a common data stream for simultaneous activation of multiple electrodes at each tile - A small mounted camera in headgear received original images - Information was extracted by image processing depending on user activity.
	Tochitsky et al. (2017)	- Light stimulation of the chemical “photoswitches” BENAQ and DEBAQ - 100 W arc lamp was used for MEA light stimulation The photon flux equivalent for BENAQ-treated retinas was calculated using 459 nm photon energy.
Cochlear implants	Fletcher and Zgheib (2020), Fletcher et al. (2020)	Calculation of haptic sound localization accuracy pre- and post-training using only haptic feedback (with varied speakers across sessions and no repetition of materials). - Conditions for localization ability: audio only, combined audio, and haptic (Audio-haptic), and haptic only Conditions were measured before and after a short training regime (15 min for each condition)
	Hillyer et al. (2019)	Assessment of auditory-visual working memory, visual working memory, and processing speed using a cognitive test battery in addition to clinical methods for speech perception.
	Távora-Vieira et al. (2018)	- Responses assessed by visual inspection and classified by presence or absence of cortical auditory evoked potential (CAEP) components - Subjects asked to use their new setting for 2–3 weeks then return for retesting. - New CAEP recordings performed during retesting to ensure new fitting maps effectively activated the auditory cortex.
	Gauer et al. (2019)	Playback devices attached to the speech processor for sessions (enhance music enjoyment for cochlear implant users)

## 5. Limitation

This paper provides an overview of the current state of closed-loop brain stimulation research and highlights its potential in the treatment of various neurological disorders such as Parkinson's disease, dementia, and depression. We also discuss the challenges of closed-loop brain stimulation, including electrode design, decoding and encoding algorithms, and the need for long-term stability and safety. Several future research directions include the development of closed-loop systems that can tailor stimulation based on multiple input signals, the use of closed-loop systems for neuromodulation of complex networks, and the integration of closed-loop systems with other systems. We also emphasize the importance of collaboration between researchers, clinicians, and patients in developing effective closed-loop brain stimulation systems that improve the quality of life of neuropathic patients.

This review has several limitations. There was heterogeneity in the comparison of closed-loop BCIs in terms of purpose, methodology, and outcome. This review mainly considered studies that involved participants in the laboratory environment, and contains scant results of work based on empirical environments because the search string did not include the words “environment,” “daily-life use,” or “company.” Additional limitations include the scope of this review. It focused more on cognitive neural BCI and less on affective BCI, particularly BCI related to emotions. Moreover, this review did not exclude animal testing or early-stage studies. Privacy of personal details concerning the users may be needed to ensure participant protection, or to comply with specified requirements for using neuroimaging in daily life. The users' subjective opinions were also not covered by this review. The articles included in this review focused on the technology used, regardless of the purpose of the research, type of tools, and the software used. Future studies should consider different types of commercial equipment to analyze differences in the impacts of each on the quality of life of the user.

## 6. Conclusion

This review examined research on the applications of brain stimulation-based closed-loop BCI systems. We broadly classified the types of stimulation into five categories: (i) DBS, (ii) TMS, (iii) tDCS, (iv) tACS, and (v) optogenetics. Overall, closed-loop BCI has exhibited the potential for improving the quality of life of patients by restoring, replacing, or rehabilitating their impaired functions as needed. Techniques of brain stimulation have the promising outcome of the capability of managing the symptoms of individuals with depression, dementia, Alzheimer's disease, and Parkinson's disease. DBS can treat mental problems when traditional therapies have failed, such as OCD. In addition, DBS is also reversible and customizable, allowing clinicians to maximize patient results by modifying stimulation parameters. Real-time stimulation adjustment allows for individualized and responsive therapy. Although pharmaceutical therapies may have systemic adverse effects, DBS has fewer and more localized side effects that may be treated by altering stimulation settings. The capacity of TMS and tDCS to alter the excitability of neurons in specific brain areas is one of their key advantages. TMS induces electrical currents in the brain using magnetic fields, whereas tDCS delivers a low current through electrodes implanted in the head. Depending on the stimulation parameters employed, these approaches can be used to stimulate or inhibit neuronal activity in specific brain regions. TMS has also been utilized to map the functional connectivity of the brain and enhance cognitive skills in individuals with traumatic brain injuries and other neurological illnesses.

By entraining neural oscillations, tACS may alter the activity of certain brain areas and improve cognitive abilities. tACS has also demonstrated potential in treating several neurological disorders, including schizophrenia and chronic pain. Optogenetics is a relatively recent approach involving the genetic modification of certain neurons to produce light-sensitive proteins. After the neurons have been changed, they may be manipulated using light given *via* implanted fiber-optic wires. Notably, optogenetics is a very recent and intrusive approach that requires genetic alteration and fiber-optic cable insertion. Before optogenetics may be utilized clinically, further study is required to establish its safety and effectiveness in people.

Such devices and applications as bionic eyes and cochlear implants can restore a significant percentage of vision and hearing. The results here show that the above areas of research have immense potential for further research. Although BCI can significantly influence the lives of people suffering from neurodegenerative and psychiatric diseases, its implementation should be preceded by a sufficiently large number of clinical trials and experiments.

This review pinpointed the different techniques of brain stimulation-based closed-loop BCI that can supplement, improve, or enhance the lives of patients. Future research in the area should seek to identify and develop more noninvasive strategies to avoid the need for surgery to reduce the risk of harm, and provide sufficient support for improving the daily lives of the affected individuals.

## Author contributions

AB and FA conceived the topic. AB conducted the conceptual design for the review. NJ and SK conducted the literature survey. AB, NJ, and SK wrote a preliminary version of the manuscript. NJ and SK contributed to selecting the articles and analyzing the results. AB and FA have supervised the work. All authors listed have made a substantial, direct, and intellectual contribution to the work and approved it for publication.
